# Electronic Transport and Quantum Phenomena in Nanowires

**DOI:** 10.1021/acs.chemrev.3c00656

**Published:** 2024-02-23

**Authors:** Ghada Badawy, Erik P. A. M. Bakkers

**Affiliations:** Department of Applied Physics, Eindhoven University of Technology, 5600 MB Eindhoven, The Netherlands

## Abstract

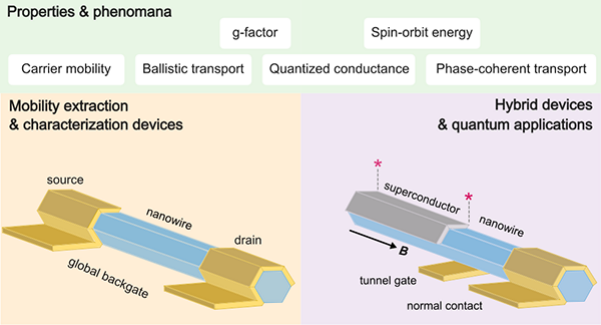

Nanowires are natural one-dimensional channels and offer new opportunities for advanced electronic quantum transport experiments. We review recent progress on the synthesis of nanowires and methods for the fabrication of hybrid semiconductor/superconductor systems. We discuss methods to characterize their electronic properties in the context of possible future applications such as topological and spin qubits. We focus on group III–V (InAs and InSb) and group IV (Ge/Si) semiconductors, since these are the most developed, and give an outlook on other potential materials.

## 1 Introduction

In the broadest sense, quantum transport describes electron flow at length scales approaching the electron’s wavelength. In this regime, many of the standard approximations in classical transport theory no longer hold, and quantum effects arise. In particular, as devices become smaller, their response no longer scales with dimensions. Consider a classical conductor, described by Ohm’s law. Its conductance is directly proportional to its cross-sectional area and inversely proportional to its length. Classically, one could envision that as the width (cross-section) of the conductor becomes smaller, the conductance decreases, tending to zero. However, as it turns out, as the width becomes comparable to the electron’s wavelength, the conductance no longer scales with width, but it is limited by a finite value of 2*e*^2^/*h*, referred to as the quantum of conductance.^1,2^

Accordingly, electrons can no longer be regarded as classically charged particles, and Ohm’s law fails to describe their wave nature. On this scale, the system enters the quantum transport regime, giving rise to quantum phenomena, such as ballistic transport with quantized resistance,^1,2^ the quantum Hall Effect,^3^ interference effects (Aharonov Bohm effect),^4^ and single electron tunneling due to the Coulomb blockade.^5^

These effects are accessible when the electrons are spatially confined by the dimensions of the sample. More specifically, when these dimensions are comparable to or smaller than three characteristic length scales: (1) the Fermi wavelength, which relates to the electron’s kinetic energy, (2) the mean free path, the distance an electron travels before its initial momentum is randomized, and (3) the phase relaxation length, the distance an electron travels before its phase is no longer correlated with its initial phase.^6^

Access to these length scales has been possible because of continuously evolving semiconductor fabrication techniques. The advent of ultrathin epitaxial semiconducting materials of unparalleled crystalline quality and purity promoted the investigation of the quantum limit of electronic transport.^7,8^ By epitaxially growing high-quality single-crystalline films of selected semiconductors with different energy band gaps, a thin layer of highly mobile electrons is created at their interface. Because electrons cannot move in the direction perpendicular to the interface, their density of states takes on discrete values, and their wave function forms a standing-wave. Conversely, electrons are free to move in the other two directions, forming the so-called two-dimensional electron gas (2DEG).

The first demonstration of a 2DEG was in a semiconductor/insulator interface, namely a silicon metal-oxide field effect transistor (Si MOSFET). Nevertheless, the GaAs/AlGaAs heterostructure is considered the prototypical example of 2DEGs.^7−9^ The lattice match between GaAs and AlGaAs yields a crystalline interface, free of scattering, leading to long electron mean free paths and mobilities exceeding 10^7^ cm^2^/(V s).^10,11^

One way to study electron transport in 2DEGs is by defining electrostatic gates on top of the heterostructure, e.g., on top of the AlGaAs layer in the GaAs/AlGaAs system. For instance, a two-gate setup depletes the electron gas underneath, thereby constraining it to a one-dimensional channel between the gates. In this setup, also referred to as quantum point contact (QPC), quantized conductance, in steps of 2*e*^2^/*h*, has been measured for the first time.^1^

Because of the flexibility of confining the underlying 2DEG with gates in any desired shape, from 1D wires to quantum dots that can host single electrons,^12,13^ 2DEGs have been the workhorse of mesoscopic transport for several decades^7−9^. However, the thin film configurations hosting 2DEGs impose severe limitations on device design. For instance, high mobility 2DEGs can only be realized in (nearly) lattice matched systems, which restricts material combinations and investigation of new materials. Moreover, to create nonplanar device geometries, the heterostructure needs to be etched, introducing imperfections in the pristine layer stack and thereby compromising device performance^14,15^.

Nanowires can overcome a number of these limitations and open up a wealth of quantum transport phenomena, as will be extensively discussed in this review. Nanowires are cylindrical nanostructures having diameters in the nanometer range (10–100 nm) and lengths that are hundreds or thousands of times longer (1–10 μm). Unlike thin films, the small nanowire footprint allows strain, due to lattice mismatch, to be elastically relaxed without dislocations. This property thus eliminates the need for expensive, lattice matched substrates. Furthermore, the nanowire geometry can flexibly accommodate new material combinations, semiconductors, dielectrics, superconductors, and magnetic materials, often epitaxially in three dimensions resulting in nanostructured devices of unprecedented complexity and functionality. The possibility to tailor material properties down to the nanoscale in nanowires, such as their composition, growth direction, crystal phase, and dimensions allows to a certain level the customization of their quantum mechanical properties and the creation of a platform to discover and probe novel phenomena in physics^16,17^.

In contrast to 2DEGs, where the electrons are confined to a conductance channel by electrostatic gating, confinement in nanowires occurs effortlessly due to their geometry. In particular, radial confinement, transverse to the flow of carriers, transpires due to the nanowire’s diameter being on the order of the characteristic length scales, Fermi wavelength, mean free path, or the phase relaxation length, leading to a one-dimensional conductance channel. Because of this natural confinement in combination with the flexibility in material combinations, nanowires have been used to explore many quantum transport phenomena, such as ballistic flow of electrons, quantized conductance, Coulomb blockade, and spin transport. In the context of quantum computing, nanowires have been used in various quantum bit (qubit) implementations ranging from superconducting-based to spin-based qubits^18−21^.

More recently, theory proposals of 2010 ignited immense interest in semiconducting nanowires as they suggested that these nanowires can be used as a platform for topological quantum computing based on Majorana quasiparticles^22,23^. In particular, by coupling a semiconducting nanowire with strong spin–orbit coupling and a large Landé *g*-factor to a conventional (*s*-wave) superconductor, such as aluminum, superconductivity is induced in the nanowire by proximity. A magnetic field parallel to the long axis of the nanowire together with the spin–orbit interaction and the *g*-factor drive the proximity-induced superconductivity to be topological with Majorana quasiparticles arising at both ends of the nanowire. One advantage of the nanowire geometry is that superconductivity can be easily induced in the nanowire, as opposed to 2DEGs, where the active transport channel is buried under an insulating layer, rendering proximity effects very challenging.

In this work we review recent progress on the growth, device fabrication, and quantum transport measurements of nanowires. In the first half of this review, we focus on group III–V semiconductor nanowires and review their synthesis methods. Moreover, we discuss a number of generic (quantum) transport phenomena that are generally used to extract their relevant parameters for various device applications, most notably quantum computing devices. Since many quantum device applications rely on semiconductor-superconductor hybrid structures, we discuss their growth and transport measurements, with again a focus on the III–V system. The second half reviews recent progress on group IV nanowires, their synthesis, transport phenomena, and devices. Finally, we conclude with an outlook section, sketching outstanding goals for nanowire research in the context of quantum transport and quantum computing.

## 2 III–V Nanowires

III–V compound semiconductor nanowires, based on group-III elements such as aluminum (Al), gallium (Ga), and indium (In), combined with group-V materials, such as phosphorus (P), arsenic (As), and antimony (Sb), are among the most researched low-dimensional systems in the field of nanoscience and technology. III–V nanowires and their alloys have been used in various applications,^24,25^ ranging from electronics^26−28^ to photovoltaics^29,30^ and photonics^31−34^. The majority of III–Vs have a direct bandgap and a high carrier mobility.^25^ Unlike group IV materials, III–Vs offer a wide range of material combinations which in turn implies that they span a wider range of properties, which can be engineered by tuning their compositions and stoichiometry.^25^ An additional degree of freedom exists for their nanowire form as they can assume different crystal structures, as opposed to their stable bulk form, thus providing an even larger range of tunable intrinsic qualities, such as band gap energies and electronic properties^35,36^.

The majority of III–V nanowires have been extensively researched for applications in photonics and optoelectronics and much less for quantum information applications^26,31,32,37−40^. Although the narrow bandgaps of indium arsenide (InAs) and indium antimonide (InSb) have rendered them suitable for infrared detection and terahertz emission applications^41−43^, they are especially useful in a plethora of quantum devices ranging from field-effect transistors^27,44,45^ to spin-based quantum bits^20,46^ due to their high carrier mobility, spin–orbit coupling, and Landé *g*-factor^47−50^. Moreover, these material properties have granted InAs and InSb front row seats to the topological quantum computing research, being model systems for the search for Majorana quasiparticles. Because of their high *g*-factor, spin-dependent phenomena can be measured at relatively low magnetic fields,^49^ which are compatible with superconductor-nanowire hybrid devices, since the superconducting properties are generally destroyed by too high magnetic fields, i.e., higher than the critical field of the superconductor.^51^ In these hybrid devices, the nanowire is predicted to be transformed into a topological superconductor with Majorana zero modes at its ends^52,53^.

### 2.1 Synthesis of III–V Nanowires

III–V nanowires can be synthesized using many techniques, that are categorized into two main ones: top-down and bottom-up. Top-down approaches start out with a bulk material, from which nanowires are carved out using a combination of lithography and etching^54,55^. While this approach results in ordered arrays of nanowires with good control over placement and density, it suffers from a few drawbacks. The challenges in overcoming the lithography limit, the difficulty of creating faceted, almost atomically flat nanowires, along with the inability to change their crystal structure and orientation has limited this top-down approach to specific nanowire device applications.^55^ Thus, far, nanowires for quantum device applications have been synthesized using the more popular bottom-up technique.

**Figure 1:**
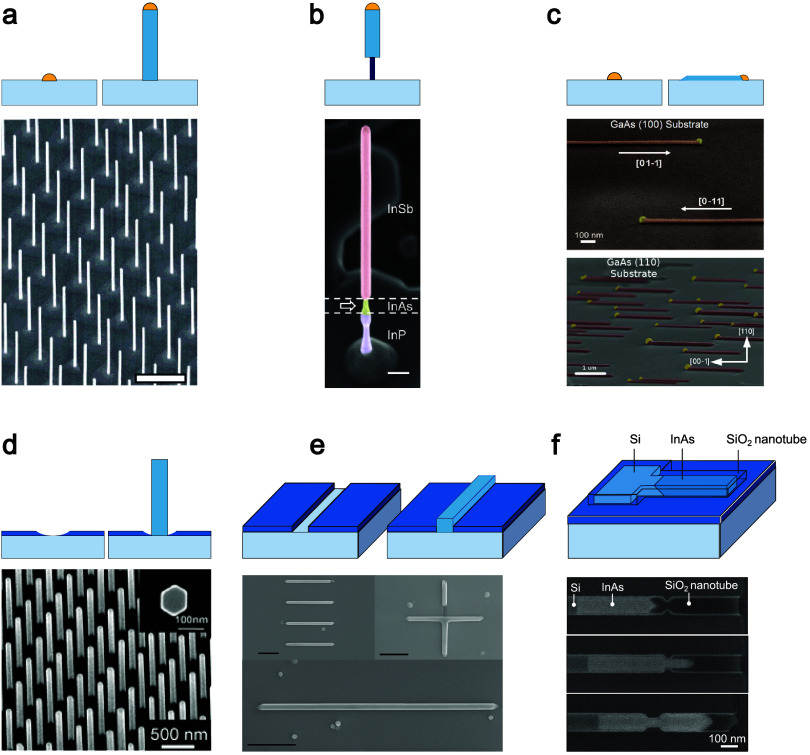
Nanowire synthesis. (a) VLS growth of InAs nanowires from gold nanoparticles. The titled SEM image shows an array of InAs nanowires (scale bar = 1 μm). (b) VLS growth of an InSb nanowire on top of a nanowire stem of different material (InP-InAs). False colored SEM of a single nanowire (scale bar: 200 nm). (c) Schematic of planar VLS nanowires. Tilted view and false colored SEM images of self-aligned planar nanowires with gold nanoparticles at the growth front. The nanowires propagate either along the [01–1] or the [0–11] direction as indicated.^56^ (d) Selective area growth of InP nanowires. The nanowires grow perpendicular to the substrate from a nanohole opening in the mask, as shown schematically. Tilted SEM image with a field of InP nanowires. The inset shows a top view of a nanowire. (a–d) Reprinted from ref (57), ref (58), ref (59), and ref (60). Copyright 2004, 2012, 2008, and 2010 American Chemical Society, respectively. (e) In-plane selective area growth. Schematics: constrictions in the amorphous mask, where the nanowires grow parallel to the substrate. Top view SEM images of in-plane selective area (110) InAs nanowires and three InAs nanowire networks on an InP (001) substrate. The orientation of the nanowires and networks are indicated. Reprinted with permission from ref (85). Copyright 2018 by the American Physical Society. (f) Template-assisted selective area epitaxy illustration. SEM images show epitaxial filling of an InAs nanowire within the silicon oxide nanotube. The InAs is epitaxially connected to the Si seed. Reprinted with permission from ref (61). Copyright 2015 AIP Publishing.

The bottom-up approach, as its name suggests, relies on the assembly of the constituent building blocks in an additive manner to create the structure, referred to within the nanowire community as “growth”. The undoubtedly most popular bottom-up technique is the vapor–liquid–solid (VLS) growth method due to its enormous flexibility. The VLS method was first demonstrated in the 1960s to synthesize silicon nanowhiskers.^62^ In VLS, metal nanoparticles, usually gold, are used to catalyze the growth, while the source materials of the semiconductor are supplied in the vapor phase. At the growth temperature, the metal particle is liquid and forms a eutectic alloy with the semiconductor. The incoming gaseous source molecules dissolve into the liquid particle until their concentration goes beyond the thermodynamic solubility limit, causing the precipitation of a solid monolayer of semiconductor material. This liquid–solid interface acts as a growth front. Since the source materials are supplied continuously, the metal particle reaches an equilibrium state, in which the solubility limit is maintained by expelling excess material as a solid, causing the nanowire to grow longer and pushing the particle upward.^63^ One of the most common metal catalysts is gold (Figure 1a). For III–V nanowires, however, the group-III material is often used as the catalyst, in the commonly known self-catalyzed VLS technique^64−66^. Noncatalytic growth of III–V nanowires, also known as selective-area epitaxy (SAE), has also been shown on masked substrates, where nanoholes are defined in an amorphous mask and act as nucleation centers for nanowire growth^67−70^. The formation of side facets with a low surface energy, which tend to grow very slowly, allows the growth to continue axially faster than radially also above the mask, resulting in free-standing nanowires^71^ (see Figure 1d).

The VLS technique and its variations, such as the vapor–solid–solid (VSS) process in which the catalyst particle remains solid during the entire growth, yield free-standing nanowires pointing along surface normal directions. For the majority of III–V nanowires, the most common out-of-plane direction is the ⟨111⟩B^72,73^. As a matter of fact, this property of nanowires has been put to use to design nanowire networks, such as crosses and T-junctions to make intricate devices, especially relevant to quantum computing applications. The first generation of such networks relied on growing nanowires on (100) substrates, where the wires will grow in one of the two available (111)B directions forming an angle of 54.7° with the substrate normal. With a 25% probability, if two neighboring wires grow in the opposite ⟨111⟩B, such that they grow toward each other, a nanowire network is formed^74,75^. The yield of such networks has been pushed to 100% by etching (100) substrates, in such a way that the two opposing (111)B planes are exposed. Gold particles positioned on these (111)B planes ensure that the nanowires always grow toward each other, forming the desired nanowire network^76−79^. Another approach to grow nanowire networks hinges on forcing the gold droplet to slide on one of the nanowire side facets and resume growth in a horizontal direction^80,81^. Although networks created using this sliding technique require fewer fabrication steps, it suffers from lower yield, as there is no preference to which facet the catalyst particle slides onto, thus further reducing the chances of merging wires.

While less common, III–V nanowires grown parallel to the substrate (in-plane growth) using the VLS method have also been reported^59,82,83^, as depicted in Figure 1c. Planar growth is generally more compatible with device fabrication. For quantum transport, free-standing nanowires and nanowire networks need to be transferred to a device substrate, before any measurements can be performed on them. This transfer process is time-consuming and not scalable. Therefore, a lot of effort has been exhausted on growing high-quality planar nanowires and nanowire networks^84−89^. These efforts tend to dispense with the catalyst particle to avoid randomness in the planar growth direction, thus ensuring high yield. Like the SAE (or selective-area growth (SAG)) approach used to grow free-standing wires, lithographically defined openings with the desired network shape or nanowire size are outlined within an amorphous mask on a crystalline substrate. Growth proceeds analogous to 2D planar growth, however selectively, only within the predefined openings (Figure 1e). Wires or networks can be grown with a large degree of design freedom, as long as the in-plane growth direction follows specific optimum crystallographic directions imposed by the substrate. These are often the low-index directions ⟨001⟩, ⟨110⟩, or ⟨112⟩, yielding flat side and top facets.^84−89^ Similar to layer growth, the choice of the host substrate becomes critical, more specifically, with regard to lattice constant and bandgap. If the lattice mismatch is too big, crystal defects within the grown nanowires become more likely. Moreover, a small bandgap of the substrate would limit the use of these wires and networks for transport experiments. In particular, it could lead to a parallel conductance channel through the substrate, making it difficult to probe the nanowire. The choice of substrate for in-plane InSb nanowires, particularly, is rather challenging, due to the large lattice constant of InSb, causing it to be lattice mismatched with all wide-bandgap III–Vs and nearly all conventional semi-insulating substrates. Yet, InSb in-plane nanowires have been grown on lattice-mismatched substrates, where the strain is relaxed very close to the nanowire–substrate interface by formation of misfits, leading to high quality InSb channels above the defected interface^87−89^. A way to mitigate lattice mismatch issues is by the use of a buffer layer, which (partly) accommodates the mismatch.

Another way to overcome this challenge and even allow in-plane growth of III–V nanowires on silicon is the so-called template-assisted selective-epitaxy (TASE) approach^61,90^. Here, the III–V precursors are guided through a silicon-oxide tube toward a small silicon seed crystal, where the III–V nanowire nucleates and subsequently grows to fill the entire template (see Figure 1f). This seed crystal can be located on either a Si wafer or a silicon-on-insulator (SOI) substrate, thus facilitating subsequent transport measurements. Although being very device compatible, this technique still suffers from challenges related to growth dynamics. In particular, the long surface diffusion in the tube toward the extremely small exposed III–V nucleus/silicon seed requires long growth times as well as causes variations in the V/III ratio within the template.^90^ A challenge for the in-plane geometries is to fabricate high quality devices. Opposite to the VLS wires, which are harvested from the growth substrate and then transferred to a device chip with, for instance, predefined gate electrodes covered with a high-quality dielectric, the device processing for the SAE wires is done afterward, limiting the thermal budget and the design flexibility. It has been proposed to grow epitaxial gates underneath the wire, but it will be challenging to realize a high-quality epitaxial surface for wire growth.

The most common growth methods, whether the nanowires are grown using VLS, SAE, are in-plane, or out-of-plane, include metal–organic vapor phase epitaxy (MOVPE), molecular beam epitaxy (MBE), and chemical beam epitaxy (CBE), each with its merits and limitations^91−95^.

### 2.2 Transport in III–V Nanowires

As discussed in section 2.1, there are various methods to synthesize III–V nanowires, each technique offering its advantages as well as shortcomings. One way to assess the quality of nanowires and to evaluate their suitability for quantum device applications is by characterizing them using transport experiments. With the transport experiment, properties such as carrier mobility, carrier density, and spin–orbit length can be extracted. Although, the nanowire quality can be evaluated based on optical properties, such as photoconductivity measurements and photoluminescence intensity^96,97^, they are outside the scope of this review and are less relevant to quantum device applications. In this section, we review some basic quantum transport experiments used to extract relevant properties for quantum device applications and quantum computing circuits in III–V nanowires with a focus on InAs and InSb.

#### 2.2.1 Carrier Mobility

Among the most basic transport experiments to characterize the electronic properties of nanowires is to determine their carrier mobility. Carrier mobility is simply a measure for how fast carriers can move through a solid under an applied electric field. Mobility is inversely proportional to the carrier effective mass and is limited by scattering events that slow down the carriers. Scattering can occur due to collision with other carriers, phonons (lattice vibrations), crystal defects, and impurities. Although mobility is rather a classical transport concept, it quantifies the level of disorder (e.g., crystal defects and impurities) within the nanowire, as it directly relates to the time between scattering events. Crystal defects in InAs nanowires have been shown to significantly reduce the electron mobility at low temperature^98,99^, which in turn could affect the device performance. We note that structural defects are more relevant for InAs nanowires because the energy difference for forming the two crystal phases, wurtzite and zinc blende, is small, requiring precise tuning of growth parameters to achieve pure phase nanowires^98,100,101^. In contrast, this energy difference is large for InSb leading to InSb nanowires free of stacking-faults with a pure zinc blende phase^58,102−104^. Nevertheless, InSb nanowires often require an indium arsenide (InAs) or indium phosphide (InP) nanowire to nucleate^58,102−104^, leading to the incorporation of unwanted arsenic and phosphorus (see Figure 1b). These foreign group V impurities can contribute to alloy scattering in InSb nanowires.

**Figure 2:**
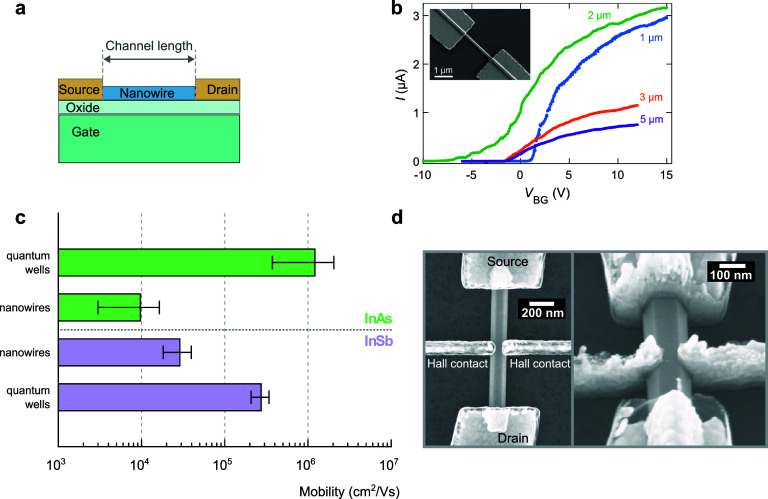
Carrier mobility and mobility devices. (a) Schematic of a typical device with the nanowire positioned on top of a substrate with an oxide layer, which acts as a global backgate. Current through the nanowire flows from the source to the drain contacts, where the source-drain spacing constitutes the channel length. (b) Pinch-off curves, i.e., current as a function of backgate voltage, *V*_BG_, and a fixed source-drain (bias) voltage obtained from devices as described in panel (a), where the channel length is specified accordingly. Inset with a top-view SEM image of a representative device.^106^ (c) Average mobility in InAs and InSb quantum wells and nanowires, which are calculated based on mobility values from various publications: InAs quantum wells^107−112^, InAs nanowires^85,98,113,114^, InSb quantum wells^15,115−118^, and InSb nanowires^58,119−121^. All averages are based on low-temperature mobility measurements. (d) SEM images top-view (left) and 35^o^-tilted (right) of a nanowire device with Hall contacts to extract nanowire mobility from Hall measurements. Reprinted with permission from ref (122). Copyright 2012 AIP Publishing.

Another great limitation for carrier mobility in nanowires is surface scattering due to their large surface-to-volume ratio inherent to their geometry. Surface scattering commonly restricts the mobility even in defect-free nanowires. Furthermore, the low effective electron mass of InSb and InAs implies a larger de Broglie wavelength, making carriers more susceptible to surface scattering compared to other materials with a high effective mass. Even more detrimental for InAs is the natural electron accumulation layer near the surface, which additionally contributes to surface scattering.^105^ In general, carrier mobility in nanowires remains at least 1 order of magnitude lower than carrier mobility in 2DEGs in thin films of corresponding materials (see Figure 2c).

To extract carrier mobility in nanowires, two main approaches are used. The first one is based on nanowire field-effect transistor measurements. Generally, this method is used more often due to its simplicity. A typical device consists of a single nanowire placed on a highly doped substrate, which acts as a global back-gate, and contacted by two metal leads (electrodes), as shown in Figure 2a. The electrodes spacing is designed to be much larger than the electron mean-free path, making the transport diffusive and classical. The conductance as a function of applied back-gate voltage (*V*_G_) is measured by applying a bias voltage typically around 1–10 mV. By fitting the pinch-off curve, i.e., the current as a function of backgate voltage (Figure 2b), the mobility can be extracted. Although this technique is relatively straightforward, there are some concerns regarding its precision in providing reliable mobility values in nanowires. These concerns are in large part related to the models used to fit the pinch-off curves to extract mobility. One limitation is the assumption that the mobility is constant within the entire conductance range, i.e., from open to pinch off, while it is also affected by the carrier density due to electron–electron interactions. Moreover, the equation used to fit the current–voltage relation assumes an ideal behavior. Therefore, any nonideal characteristics including kinks in the pinch-off curves or hysteresis could lead to erroneous estimations of the carrier mobility^123,124^. Another limitation is the uncertainty in the capacitance value between the nanowire and the gate, which is used in the fitting equation for determining the mobility, in turn affecting the extracted mobility values. The experimental extraction of the capacitance is rather challenging due to the extremely small dimensions of the nanowire^98,114,119^. This issue becomes even more critical for top-gated devices because of changes in capacitance values within the measurement range and higher sensitivity for variations in gate-oxide thickness.^125^ Moreover, depositing a gate oxide can strain the nanowire or change its surface properties, thus introducing even more uncertainty to this measurement technique.^125^ In-plane nanowires are plagued by this top-gated geometry because of the difficulty to back-gate semi-insulating substrates.

Despite limitations, FET measurements remain among the most common techniques to extract carrier mobility in nanowires^58,98,114,119,120,126,127^. Developing methods to measure nanowire-device capacitance and designing models to better evaluate it are amidst the efforts in minimizing uncertainty in extracted mobility values^128−130^.

The second approach for measuring mobility in nanowires is by extracting it from the Hall Effect. This approach has the advantage that it does not require the determination of the nanowire-device capacitance. Nevertheless, the fabrication of a Hall bar by creating side-contacts (Figure 2d) is challenging since it requires accurate alignment and entails the nanowire diameter be sufficiently large to be able to accommodate contacts^122,131^. Another challenge is the susceptibility of the side-contacts to cross-talk triggered by the small nanowire diameter. In this Hall Effect geometry, the side-contacts are used to measure the Hall voltage or resistance^122,131,132^. The advantage of using side-contacts on a single nanowire is the possibility to directly probe the nanowire resistivity, where the four-probe measurement allows for the exclusion of the contact resistance. For a fixed back-gate voltage and a source-drain current, the Hall voltage (*V*_H_) is measured as a function of magnetic field. From the slope of the Hall voltage curve, the electron density *n* can be obtained. The mobility (μ) can be calculated from the carrier density (*n*) and resistivity (ρ); 1/ρ = *neμ* with *e* the charge of an electron.

Instead of using side contacts for the Hall Effect device configuration, two crossed nanowires, i.e., a nanowire cross junction can be used, such that current passes through one nanowire, while the voltage drop (the Hall voltage) is measured across the other.^77^

Once the mobility and carrier density are known, the electron mean free path *l*_mfp_, the average distance an electron travels between two scattering events, can be estimated. Due to the large surface-to-volume ratio of nanowires, electrons experience more scattering events at the nanowire surface than in 2-dimensional electron gases (2DEGs), where the quantum wells are protected by epitaxial layers, keeping the carriers well beneath the surface. Methods aimed at reducing contributions from low mobility surface-scattered electrons include passivation and protection of the nanowire surface^133,134^. Gül et al. show that carrier mobility can be improved by evacuating the sample space long enough to reduce surface adsorbates on the nanowire device.^119^ Furthermore, passivating InAs nanowires with the higher bandgap indium phosphide (InP) in the form of a few nanometer thick epitaxial shell has been shown to enhance electron mobility by a factor of 2^133,134^. While passivation leads to improvement in mobility for InAs nanowires, passivating InSb nanowires with a few nanometers thick epitaxial, lattice-matched cadmium telluride (CdTe) shells showed no change in mobility.^106^ These results invite the questions, whether thicker shells are required to achieve improved mobility in InSb nanowires, the process of removing the InSb surface oxide prior to the shell deposition is the limiting factor, or rather a combination of both. Gooth et al. rely on TASE to embed their InAs nanowires into a silicon oxide template already during growth, thus suppressing the InAs surface oxide, a major scattering source, and show an electron mean free path close to one micron, several times longer than previously reported for InAs nanowires.^135^

Despite these efforts, carrier mobility in nanowires remains much lower compared to their 2DEG counterpart (see Figure 2c). As shown in Figure 2c, low-temperature mobility in InSb quantum wells averages around 0.3 × 10^6^ cm^2^/(V s)^15,115−118^ compared to an average of 3.0 × 10^4^ cm^2^/(V s) for InSb nanowires^58,119−121^. Similarly, the average low-temperature mobility in InAs 2DEGs is 1.2 × 10^6^ cm^2^/(V s)^107−112^, while the average mobility in InAs nanowires lies at an astounding 1.0 × 10^4^ cm^2^/(V s)^85,98,113,114^.

In addition to surface scattering, extrinsic disorder introduced during device fabrication can also affect the electronic properties of nanowires. For instance, physical etching methods, e.g. using argon ion bombardment, to remove the nanowire surface oxides before depositing the metal electrodes to contact the nanowire can cause damage to the nanowire and thus introduce sources of scattering. Chemical etching can selectively etch away the oxides and is therefore less harsh and less damaging to the nanowire, as reflected in electron transport measurements^136,137^. A common etchant that works well for both InSb and InAs nanowires is ammonium polysulfide (NH_4_)S_*x*_, as it is self-terminating and etches slowly and uniformly while protecting the surface against reoxidation for the time required to evaporate the metal contacts^137−139^.

Beside electron mobility and carrier density, there are other properties that determine the suitability of nanowires for given device applications. While for transistors mobility is considered a key metric, for Majorana-based devices mobility is merely a measure of the amount of disorder in the nanowire and properties, such as when strong spin–orbit coupling and a large Landé *g*-factor are necessary.^140^ Transport experiments that are used to extract these and other properties can generally be classified in diffusive or ballistic transport depending on the channel length of the given device. In particular, diffusive transport dominates when the channel length is much larger than the electron mean free path, whereas ballistic transport takes place when the channel length is smaller than or comparable to the electron mean free path.

#### 2.2.2 Ballistic Transport, Quantum Point Contacts, and *g*-Factor

**Figure 3:**
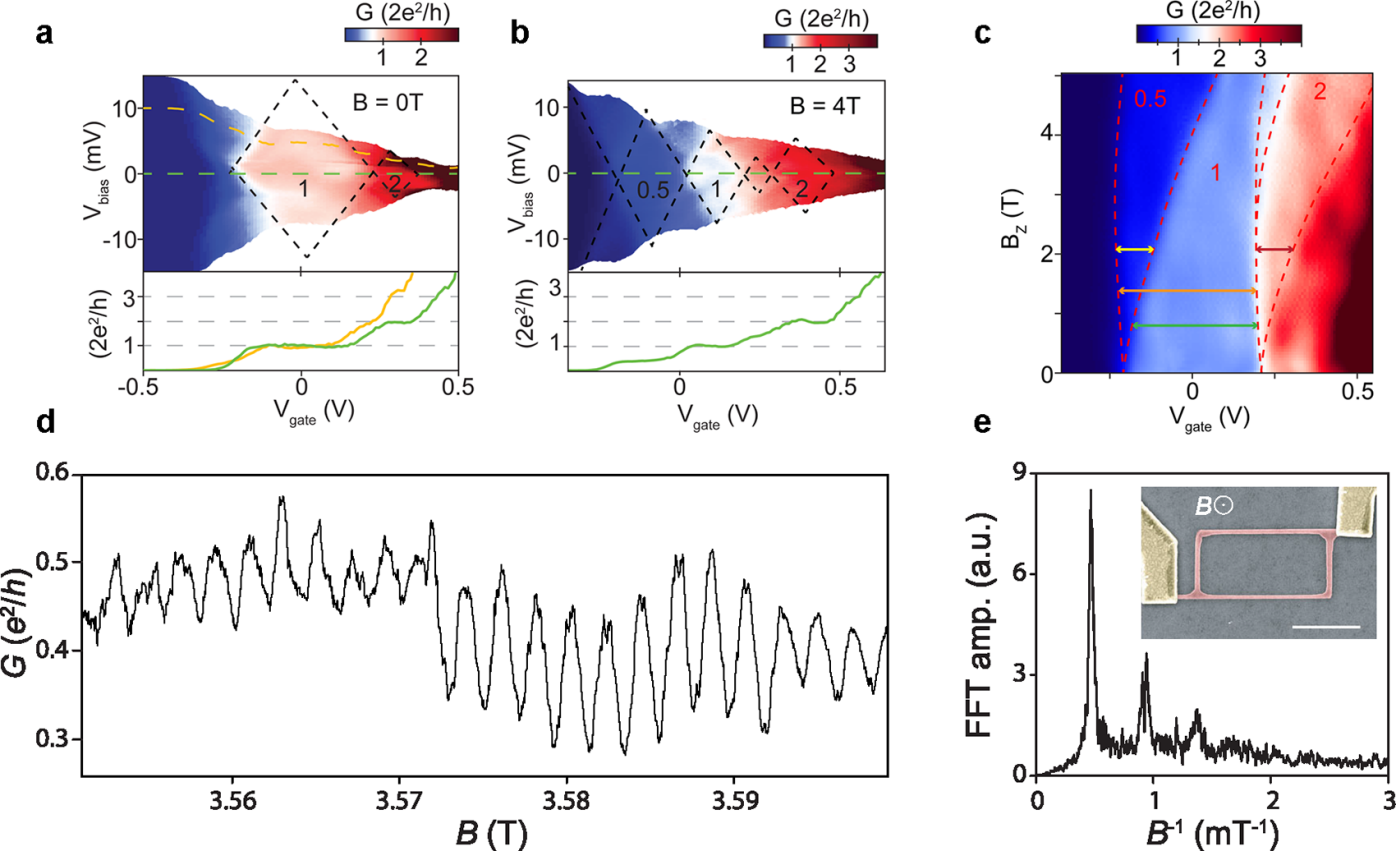
QPC, *g*-factor, and coherent transport measurements. (a) QPC device measurements, showing a differential conductance (*G* = d*I*/d*V*_bias_) map as a function of *V*_bias_ and *V*_gate_ at magnetic field *B* = 0 T and (b) an out-of-plane magnetic field *B* = 4 T. Black dotted lines indicate regions of constant conductance. Differential conductance line cuts taken at (a) 0 and 10 mV and (b) 0 ± 0.2 mV bias voltage are shown in the bottom panel, respectively. (c) Differential conductance as a function of out-of-plane magnetic field *B*_*z*_ and *V*_gate_ taken at zero bias voltage. Red dashed lines serve as a guide to the eye and indicate the sub-band spacing. The conductance plateaus 0.5, 1, and 2 are indicated. Fitting the energy level spacing *E*_1*↓*_ – *E*_1*↑*_ (separated by the yellow horizontal arrow) yields the *g*-factor of the first sub-band *g* = 39 ± 1.^137^ (a–c) Reprinted from ref (137). Copyright 2016 American Chemical Society. (d) Magneto-conductance of the device shown in the inset in (e) with Aharonov–Bohm oscillations with a period of ≈2 mT. (e) Fast Fourier Transform (FFT) spectrum of the Aharanov Bohm oscillations shown in panel (d), showing frequency peaks up to the third-order harmonic. Inset: false-colored SEM image of the device. An InSb nanowire loop (red) with metal contacts of chromium/gold (yellow). Scale bar is 1 μm. An out-of-plane magnetic field is applied, and measurement is performed at a temperature of 20 mK. The measured Aharonov Bohm period matches the loop area of ≈2 μ m^2^.^89^

In confined systems such as a one-dimensional nanowire, electrons are limited to discrete and quantized energy levels. As a function of an external gate voltage, the conductance varies in discrete steps of 2*e*^2^/*h*, corresponding to the (de)population of one-dimensional sub-bands^2,136,137,141^. Specifically, as the gate voltage is varied, the Fermi level is swept giving access to new sub-bands resulting in conductance plateaus. In order to increase the likelihood of observing these plateaus, relatively short nanowire segments are considered, such that electrons can travel ballistically through the channel and scattering is reduced. Measurements on short segments are achieved by forming constrictions in the nanowire, so-called quantum point contacts (QPCs). In such a configuration contacts are placed very close together, 150 to 300 nm, while the nanowire is depleted by a nonlocal back gate, or alternatively, by using longer nanowire segments in combination with a local top gate^136−138^. Differential conductance (*G* = *dI*/*dV*) as a function of bias voltage and gate voltage resolves a diamond for each plateau (Figure 3a,b). Within each diamond the conductance is fixed to its quantized value.

Generally, conductance quantization is rather difficult to achieve in semiconductor nanowires due to scattering events along the transport channel (typically a few hundred nanometers long), possibly induced by crystal imperfections or surface states disrupting the uniformity of the electrostatic environment. Such scattering events together with the radial confinement increase the probability of backscattering, the reflection of electrons back to the metallic leads, which further smear out the conductance plateaus. Accordingly, a strong magnetic field is typically applied (>4 T) in order to suppress electron backscattering, leading to well-defined conduction plateaus^120,136^. Albeit conductance plateaus have been measured in both InSb and InAs nanowires at zero magnetic field^135,137,142,143^.

Applying a magnetic field, *B*, splits the spin-degenerate sub-bands by the Zeeman energy *E*_Z_ = *gμ*_B_*B*, from which the *g*-factor can be extracted with μ_B_ as the Bohr magneton and *g* the Landé *g*-factor, a value which gives insight on the intrinsic spin properties of the system. This lifting of the spin-degeneracy results in half integer conductance plateaus appearing at multiples of *e*^2^/*h*, with half-sized diamonds for each plateau (Figure 3b). Measuring conductance quantization as a function of source-drain voltage and magnetic field (sub-band spectroscopy) additionally enables the determination of the sub-band spacing, see Figure 3c. Studies to estimate the sub-band spacing in InSb nanowires with a diameter of 70–90 nm yielded values around 15 meV^136,137^. In InAs nanowires, however, the sub-band dispersion in a magnetic field is heavily influenced by Fermi level pinning^144,145^, which gives rise to a surface accumulation layer leading conduction to occur close to the nanowire surface^146,147^. The extracted *g*-factor for InSb nanowires is on average 38, while values as high as 55 have been measured^49,120,137^. For InAs nanowires, the extracted *g*-factor tends to be a bit lower, in the range of 2–18^47,141,148,149^.

#### 2.2.3 Phase-Coherent Transport and Weak Antilocalization

Nanowire devices, whose dimensions are larger than the electron mean free path, are dominated by diffusive transport. Within this diffusive regime, if most of the scattering events are elastic then the phase information on the electron is preserved, implying that the phase coherence length is comparable to the device size. This phase information can be extracted from quantum interference measurements, such as the Aharonov-Bohm effect. The Aharonov-Bohm effect,^151^ a quantum mechanical phenomenon, occurs when electrons orbit a ring enclosing a magnetic flux, where the ring dimensions are comparable to or smaller than the phase coherence length. The moving electrons acquire a phase because of the enclosed magnetic flux. This phase can be computed by integrating the vector potential along the path that the electron traverses. Since electrons accumulate a different phase depending on which arm of the ring they take, they interfere constructively or destructively depending on their relative phase difference. The resulting interference pattern shows periodic oscillations, a characteristic signature of the Aharonov-Bohm effect, with one period corresponding to one magnetic flux quantum (ϕ_0_ = *h*/*e*), where *h* is Planck’s constant and *e* the electron charge.^152^

Aharonov-Bohm measurements have been performed on nanowire networks in the form of loops (Figure 3d,e), and a coherence length as high as 13 μm has been extracted^85,86^. Beside the nanowire loop structures, the Aharonov-Bohm effect can be measured on the nanowire surface in core–shell nanowire configurations, more specifically, in material combinations where the core is resistive and the shell is conductive, such as GaAs-InAs core–shell nanowires. Applying a magnetic field along the nanowire axis yields an oscillatory behavior in conductance as a function of magnetic field, analogous to the Aharonov-Bohm effect in nanowire networks^25,153−158^. In general, the phase coherence length tends to be much longer than the nanowire shell circumference. Moreover, Aharonov-Bohm oscillation measurements are used to study surface-states in topological insulator nanowires, materials with an insulating core/bulk and a conducting surface^159−161^. These measurements can identify the topological nature of these surface states.

A phenomenon commonly observed in quantum transport measurements within this diffusive regime is the so-called weak antilocalization (WAL) effect. Essentially, at low temperatures in a disordered system elastic electron scattering, e.g., with impurities, prevails over inelastic scattering with lattice vibrations (phonons), for example. The phase interference between pairs of back scattered electrons following time-reversed paths is constructive. More specifically, due to the identical length of both paths, the accumulated phases are identical, leading to constructive interference, which lowers the conductance, known as weak localization. In systems with strong spin–orbit coupling, such as InSb and InAs, the spin couples to the momentum and rotates in the opposite direction depending on the electron’s direction (forward versus backward). Thus, spin–orbit interaction contributes an additional phase which leads to destructive interference and higher conductance. This destructive interference is referred to as weak antilocalization. An applied magnetic field destroys this weak antilocalization effect, resulting in a conductance peak at zero magnetic field. By fitting the peak curvature, the spin–orbit strength can be extracted. Weak antilocalization magneto-conductance measurements have been performed on both InAs and InSb nanowires, and they provide a direct indication of the nanowire’s spin–orbit strength^50,162−169^. Although, InSb and InAs nanowires are known to have among the highest values for spin–orbit energies, the spin–orbit interaction for certain compositions of InAs_*x*_Sb_1–*x*_ has been shown to exceed the values for both InAs and InSb^25,170^.

#### 2.2.4 Quantum Dots

**Figure 4:**
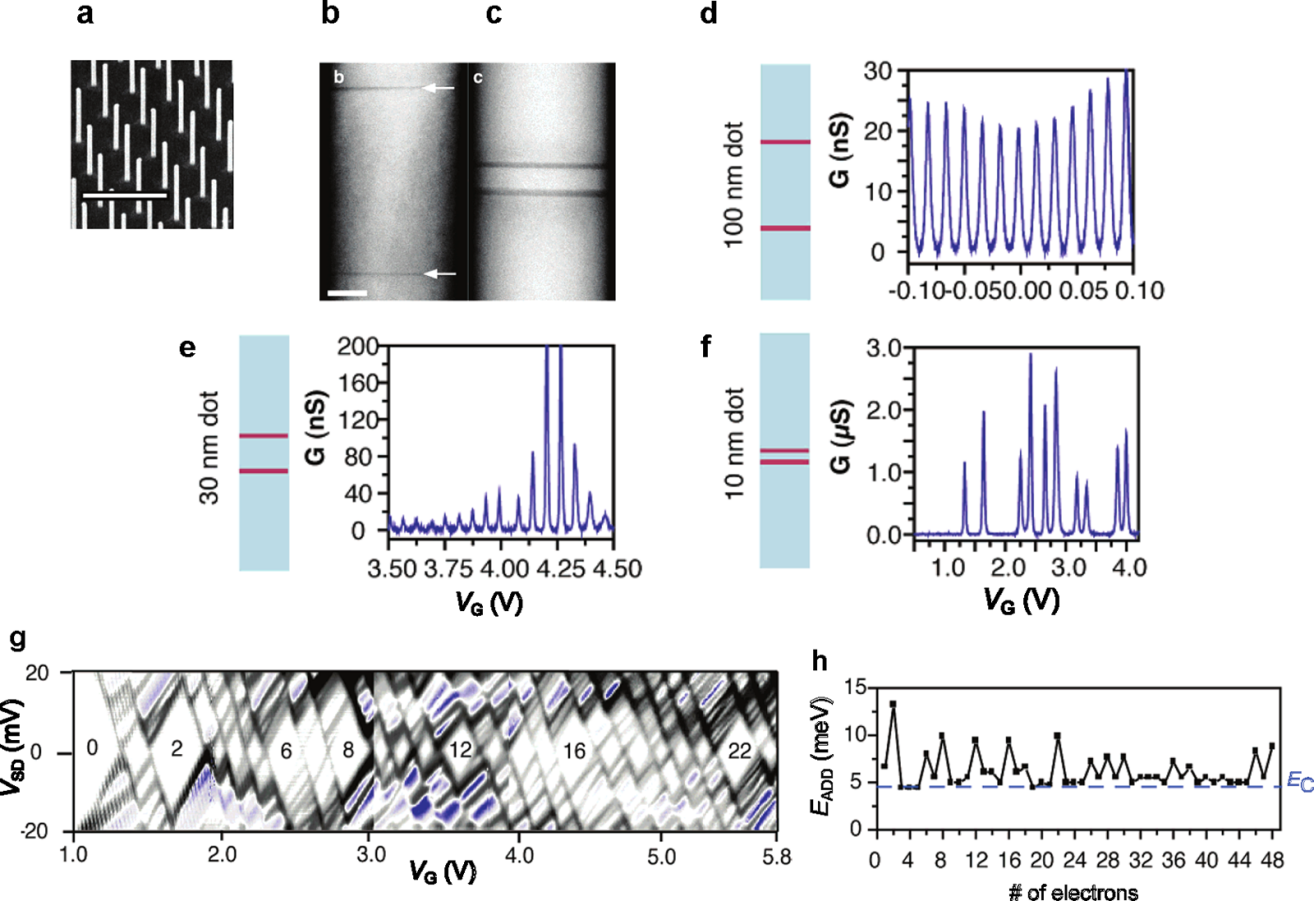
Few electron quantum dots. (a) SEM image of an array of InAs nanowires. Scale bar is 1 μm. (b and c) Dark-field scanning transmission electron microscopy (STEM) images of a nanowire with a 100 nm long and a 10 nm long InAs quantum dot, respectively, between two thin InP barriers. The InP barrier thickness is 3 and 3.7 nm, respectively. Scale bar is 20 nm. (d–f) Conductance *G* as a function gate voltage *V*_G_ for differently long InAs quantum dots, as labeled. (d) Oscillations are periodic. (e) For the 30 nm dot, the level spacing at the Fermi energy is comparable to the charging energy, and the Coulomb oscillations are no longer periodic. (f) The 10 nm dot is depleted of electrons at zero gate voltage. Increasing the gate voltage adds electrons one by one. For some electron configurations, the addition energy is larger corresponding to filled electron shells. Measurement taken at 4.2 K. (g) Differential conductance (d*I*/d*V*) as a function of bias (*V*_SD_) and gate voltage with Coulomb diamonds. (h) Addition spectrum of the device in panel (g) with the charging energy *E*_C_ nearly constant for all gate voltages.^150^ (a–h) Reprinted from ref (150). Copyright 2004 American Chemical Society.

Another common way to extract spin properties and *g*-factor is using a quantum dot configuration. Quantum dots are small conducting islands with a discrete set of electronic energy levels. The spacing between these energy levels increases as the quantum dot size decreases (see Figure 4). In quantum dot devices, electrons can tunnel one at a time onto the quantum dot (island) via tunnel junctions from metallic leads. The net charge on the island is controlled with a gate electrode, which periodically switches the state of the island between a conducting and a current-blocking state^171,172^. The current-blocking state occurs due to Coulomb blockade and manifests as Coulomb diamonds in differential conductance maps as a function of bias voltage and gate voltage (see Figure 4g). Generally, quantum dot devices need to be operated at very low temperatures, such that the thermal energy of the electrons is much smaller than the charging energy of the island. In nanowires, quantum dots can be created by confining a nanowire segment between two axially grown tunnel barriers, for instance an InAs quantum dot confined between indium phosphide (InP) barriers^150,171^, as shown in Figure 4. Alternatively, they can be created by defining a nanowire segment between metallic leads, where the nanowire segment is isolated from the leads by tunnel barriers. These tunnel barriers can be constructed either by using local gates to locally induce barriers^173−175^ or by ensuring the metallic leads connect to the nanowire segment via Schottcky barriers, instead of Ohmic contacts^49,176^.

Magnetotransport measurements allow the extraction of the *g*-factor for individual energy levels of the quantum dot. Quantum dots in InSb nanowires have shown *g*-factors as high as 70.^49^ While *g*-factors extracted from InAs nanowire quantum dots are much lower (≈18), they still exceed the bulk *g*-factors of InAs which are roughly 15.^148^ Quantum dots offer a notable platform to explore low-dimensional physics phenomena to study single electrons and manipulate the spin of single electrons. In fact, the possibility to initialize and readout the spin of single electrons confined in quantum dots renders this system a viable candidate for spin qubits^165,177,178^.

Spin qubits have been demonstrated in quantum dots defined in InAs^20^ and InSb^165^ nanowires, nevertheless, their spin coherence time is relatively short-lived compared to spins in silicon quantum dots^179,180^ because of the nearly absent magnetic nuclear spins in silicon, as discussed in Section 3. Moreover, the tunability of quantum dots including their geometry, energy spectrum, and the ability to couple multiple dots enables the manipulation of electronic states,^177^ where quantum dots have been shown to serve as tunable charge and energy filters.^181^ More recently, coupled quantum dots in an InSb nanowire in combination with a hybrid semiconducting-superconducting segment have been used to realize a Kitaev chain, potentially opening a novel platform to study Majorana physics.^182^

#### 2.2.5 Extracted Properties in Nanowires vs Bulk

The discussed experiments used to extract the different properties, ranging from carrier mobility through to spin–orbit strength, reflect that for most of the properties the bulk and the nanowire geometries are comparable. In some cases, such as the spin–orbit strength, nanowires even outperform their bulk counterpart^183,184^. Nevertheless, there is a huge discrepancy between mobility values in bulk compared to nanowire structures. This large discrepancy bids the question what is the limiting factor for mobility in nanowires. Is it surface scattering, and if so, can it be addressed by adding thick, defect-free shells around the nanowires? Is the mobility limited by damages introduced during device fabrication processes, for instance by the oxide removal step? Are in situ device fabrication methods required to bypass the oxide removal step and result in less damaged wires? These open questions can only be addressed by an iterative approach including nanowire growth, device fabrication, and transport measurements.

### 2.3 Fabrication of Superconductor-Nanowire Hybrids

Superconductor-nanowire hybrids are defined as nanostructures made of a nanowire covered with a superconducting layer on all or some of the nanowire facets typically referred to as full shell or partial shell, respectively. When it comes to superconductor-semiconductor nanowire hybrids, extensive research has been devoted toward developing methods to selectively deposit the superconductor on the nanowire. These methods are aimed at minimizing device fabrication steps and keeping the nanowire–superconductor interface as pristine as possible. On the one hand, the atomic scale uniformity of nanowire–superconductor interface turns out to be of paramount importance to the induced superconducting properties^53,185−187^. On the other hand, these selective deposition techniques enable the investigation of various superconductor materials and semiconductor-superconductor material combinations, since they dispense with postdeposition etching steps that are not always selective and could damage the heterostructures^78,188,189^.

Before the development of these selective deposition methods, the first generation of hybrid devices made use of conventional fabrication methods, where the native oxide of the nanowire is removed, followed by sputter-deposition or evaporation of the superconductor^190−192^. Eliminating the native oxide surrounding the nanowire is essential to ensure proper electrical contact with the superconductor. Similar to FET nanowire devices, the native oxides are removed either by bombardment of ionized argon atoms or by wet chemical etching, typically for InAs and InSb, using an ammonium polysulfide solution. These processes may result in a roughened surface and nonideal interfaces.

In order to minimize or even eliminate device fabrication steps, cutting-edge techniques have been developed to deposit high-quality superconductor shells with immaculate, uniform, and, in some cases, epitaxial interfaces to the nanowire. The seminal paper of Krogstrup et al. introduced concepts that enabled epitaxial superconductors (aluminum) on (InAs) nanowires with homogeneous and uniform interfaces exhibiting considerably improved superconducting and transport properties compared to traditional fabrication techniques.^186^ These concepts include depositing the superconductor at cryogenic temperatures as well as keeping the nanowires in vacuum throughout the entire process from the nanowire growth up to the superconductor deposition, commonly referred to as *in situ* deposition. Low temperature deposition limits the diffusion of the superconductor and dewetting, i.e., agglomerating into isolated islands, thus promoting uniform superconductor layers. Keeping the nanowires in vacuum ensures the nanowire surface remains oxide-free, thereby fostering a pristine, homogeneous superconductor–semiconductor interface and allowing good electrical contact between the superconductor and the nanowire. Another important requirement is the capping of the superconductor layer while it is still cold to protect it and secure it from dewetting as it warms up to room temperature. The capping, which also takes place in situ, is either done by exposing the superconductor to a flow of oxygen to form a self-terminating oxide layer around the superconductor or by depositing an oxide-based capping layer. The concepts outlined in the paper of Krogstrup et al. motivated a lot of research in other material combinations, such as InAs with lead (Pb), tantalum (Ta), niobium (Nb), and vanadium(V) as well as InSb with aluminum (Al), Pb, and tin (Sn)^78,193−195^.

In-situ processes are not always possible, and the vacuum has to be broken after nanowire growth for nanowires grown in a system that is not attached to the superconductor-deposition chamber or for transferred nanowires next to nanowalls for selective superconductor deposition. In these cases, quasi-in situ approaches are adopted, where the nanowire surface oxides are removed in the same system as the deposition of the superconductor. These approaches generally use atomic-hydrogen in high-vacuum systems to gently clean the nanowire from surface oxides after which the nanowires are transferred to the deposition chamber without breaking vacuum, hence *quasi*-in situ. Quasi-in situ techniques yield smooth and uniform superconductor–semiconductor interfaces comparable to in situ techniques and do not compromise device performance^187,194,196^. While in situ deposition of the superconductor has significantly increased the superconductor–semiconductor interface quality, the superconductor needs to be partly removed afterward in order to make a functional device. These etching steps may be detrimental to the transport properties of the most important segment of the device, the semiconductor nanowire between the superconductor and a normal metal.

**Figure 5:**
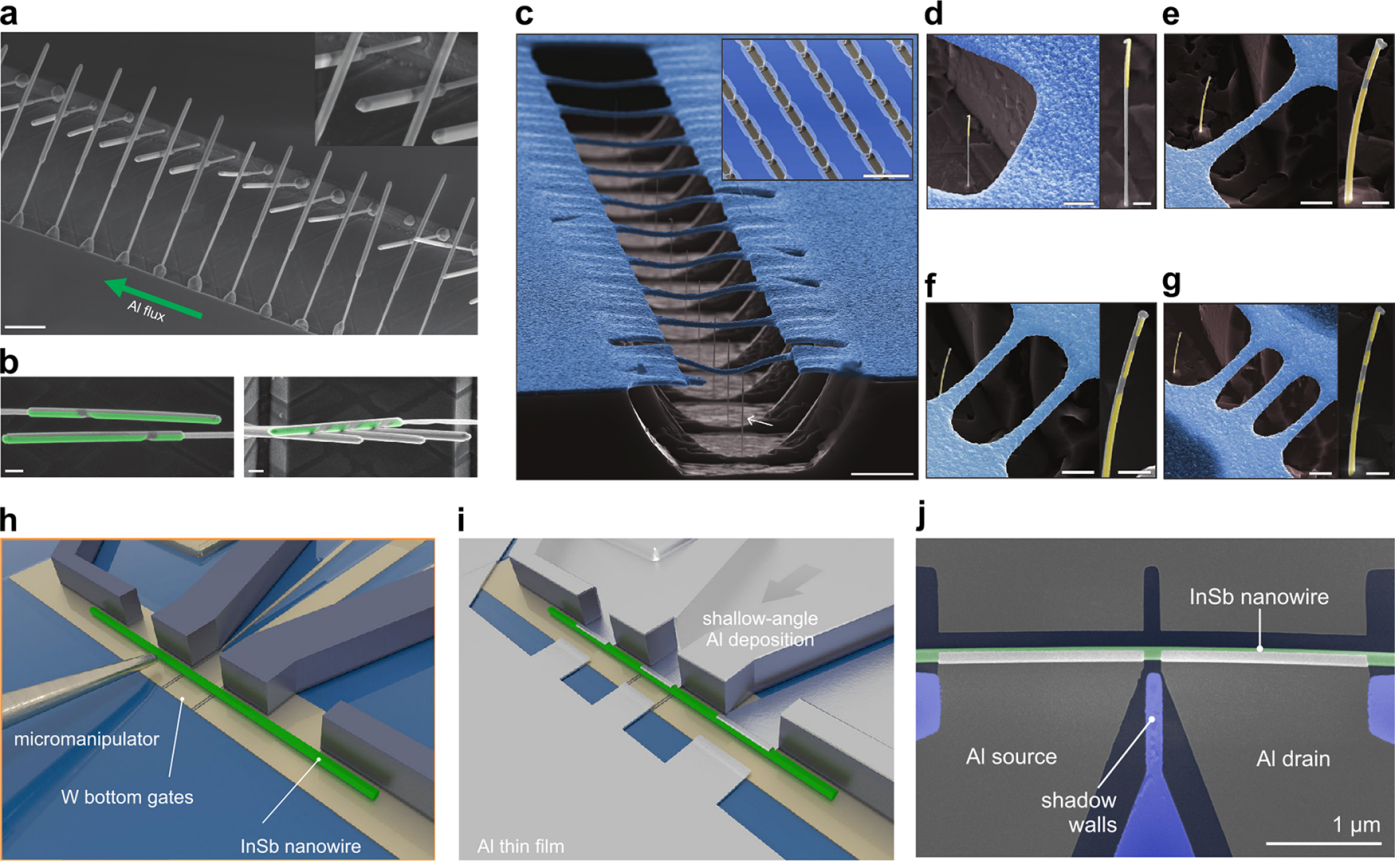
Selective deposition schemes. (a) An SEM image, taken at a 45^o^-tilt, of an array of InSb nanowires grown on a trench. The green arrow indicates the direction of Al beam flux during the superconductor deposition (Scale bar: 1 μm). Inset: magnified area showing crossing wires, barely touching. Each InSb nanowire is covered by two Al segments separated by a shadowed region. The number of shadows is determined by the number of crossing wires in front of the shadowed wire. (b) False-colored SEM images of InSb nanowires with two (left) and four (right) Al islands (scale bar: 200 nm).^79^ (c) False-colored SEM of SiO_*x*_ (blue) bridges suspended across an InAs trench (gray). InAs nanowires are grown in the trench in proximity to the bridges, the shadow mask (Scale bar: 5 μm). Inset: Overview of the sample (scale bar: 50 μm). (d–g) False-colored SEM images of the shadow-deposited nanowires, where yellow indicates the superconductor and gray the bare InAs nanowire with various selective-deposition geometries: (d) half-shadowed, (e) Josephson junction, (f) island, and (g) double-island. Scale bar: 2 μm and for the single nanowire inset: 500 nm.^188^ (h) Schematic: transfer of the nanowires with a micromanipulator onto local bottom gates (covered by Al_2_O_3_ dielectric) close to the Si_3_N_4_ shadow walls. (i) Illustration of a device after Al deposition at a shallow angle. (j) False-colored SEM image of an InSb nanowire (green) Josephson junction with (gray) Al as the superconductor.^187^

This challenge has been solved by selective deposition methods, which rely on the directionality of evaporation, specifically the directionality of the evaporated beam of superconducting material to shadow deposit the superconductor (see Figure 5). In particular, when two structures are placed behind one another, the front structure, i.e, the one facing the beam of material, casts a shadow on the structure behind it thereby blocking the evaporated beam. Areas on the back-positioned structure falling outside the shadowed region get deposited with the superconductor, hence selective deposition. This shadowing technique enables the patterning of the superconductor on the nanowire during its deposition, thus dispensing with fabrication steps, such as lithography and etching, which could compromise the device properties and performance. Numerous schemes have been devised to enable shadow deposition of superconductors on nanowires, where the shadowing objects range from nanowires grown on etched trenches^78^ (Figure 5a,b, short out-of-plane nanowires,^193^ nanoflakes (nanoflags)^194,197^, suspended silicon-oxide nanobridges^188^ (Figure 5c–g), all the way to patterned and grown nanowalls^187,189^. For the majority of these techniques, the shadow deposition takes place on the growth chip, which means that after deposition the nanowires still need to be transferred to the device chip where minimal fabrication steps are required to create the final device. The use of nanowalls as shadowing objects, however, has been mainly developed for the device chip (Figure 5h–j). In particular, the nanowire is transferred or grown in-plane next to a wall on the device chip prior to the shadow deposition, such that after the superconductor deposition the device is complete and no further fabrication steps are required^187,189,196^. The elimination of fabrication steps in hybrid devices using transferred VLS nanowires in combination with shadow-wall deposition have yielded significant improvements in transport properties, device quality, and reproducibility^187,196^. These results highlight the fragility of these hybrid devices and underline how crucial it is to preserve the pristine and homogeneous superconductor–nanowire interface.

#### 2.3.1 Hybrid Nanowire Devices

**Figure 6:**
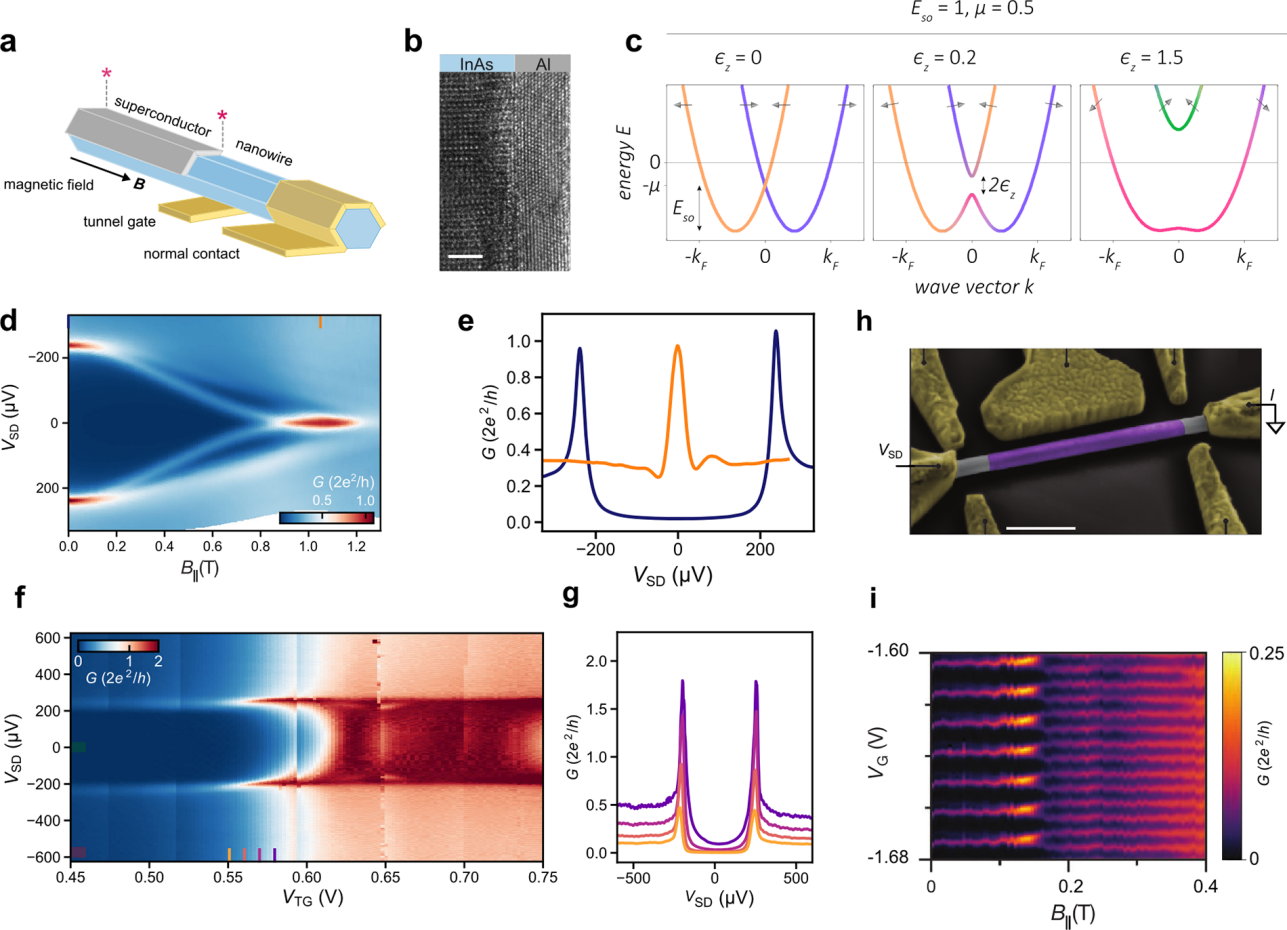
Hybrid devices. (a) Schematic of a tunneling spectroscopy device: a nanowire in a magnetic field, contacted by a superconductor and a normal metal. The noncovered section is electrostatically controlled by a bottom tunnel gate electrode. Majoranas are expected at the two ends of the hybrid region (asterisks).^203^ (b) TEM image of an epitaxial InAs/Al interface taken along a cut made perpendicular to a nanowire facet (scale bar: 2 nm), courtesy of Martin Bjergfelt and Jesper Nygård. (c) From left to right: dispersion relation in a nanowire with strong spin–orbit coupling *E*_SO_ = 1. A magnetic field introduces a Zeeman energy ϵ_*z*_ = 0.2 and opens a gap in the spectrum at the crossing point (*k* = 0) along with a smooth spin evolution along the bands. Increasing ϵ_*z*_ increases the Zeeman gap and polarizes the spins more along the external magnetic field.^204^ (d) Differential conductance as a function of bias voltage, *V*_SD_, and magnetic field, *B*_∥_. (e) Line-cuts of from panel (d) at *B* = 0 T (blue) and at *B*_∥_ = 1.05 T (orange), exhibiting a zero-bias conductance peak.^187^ (f) Differential conductance, *G*, as a function of source–drain voltage, *V*_SD_, and bottom tunnel-gate voltage, *V*_TG_, resolving a hard induced superconducting gap. The super gate, which controls the chemical potential of the hybrid nanowire segment, is grounded.^187^ (g) Line cuts taken from panel (f) at the positions indicated by the colored lines. (h) False-color SEM of a Majorana island device with an Al (purple) island and an InAs (gray) nanowire (scale bar: 500 nm).^188^ (i) *G* as a function of *B*_∥_ and gate voltage for a Majorana island showing a series of 2e-periodic Coulomb peaks below about 150 mT and nearly 1e-periodic peaks above about 150 mT.^188^

Extensive research in creating superconductor–semiconductor nanowires with immaculate interfaces is driven in large part by potential applications in topological quantum information processing. In particular, in 2010, theory proposals postulated that topological superconductivity with Majorana quasiparticles, building blocks for topological quantum computers, can be engineered from well-known, conventional components^22,23^. By combining a semiconducting nanowire with strong spin–orbit coupling and a large Landé *g*-factor, e.g., InSb and InAs, with an *s*-wave superconductor, a topological superconducting phase can be engineered (Figure 6a–c). Proximity between the superconductor and the nanowire induces superconductivity in the nanowire, yet, of an unconventional nature.^52^ As opposed to the common *s*-wave pairing in conventional superconductors, the interplay between the spin–orbit coupling, the induced superconductivity, the Fermi energy of the nanowire, and the Zeeman splitting due to an applied magnetic field, results in *p*-wave Cooper pairing and converts the nanowire into a topological superconductor with Majorana quasiparticles at its ends^22,23,198^. The detection of these Majoranas is challenging because of their zero charge and energy. Nevertheless, electrical measurements relying on tunneling spectroscopy from a normal conductor into the superconductor are expected to resolve a state at zero bias voltage (energy). Essentially, a normal metal-nanowire-superconducting (N-NW-S) junction device is used, where the N serves as the tunneling probe. The nanowire segment is depleted by a local bottom gate and serves as a tunnel barrier between the N and S (Figure 6a). Differential conductance measurements *d*I/*d*V versus bias voltage constitute a spectroscopic measurement of the density of states, where a Majorana is expected to appear as a conductance peak at zero bias (Figure 6d,e). The first signature of Majoranas was first detected in 2012 in an InSb nanowire coupled to a niobium titanium nitride (NbTiN) superconductor.^190^ This result by Mourik et al. instigated enormous curiosity and led to a vast amount of research papers attempting to increase the height of the Majorana peak to the theoretically predicted 2*e*^2^/*h* value and reduce the density of states within the induced superconducting gap, the soft gap problem.

The soft gap problem is mostly attributed to disorder in the superconductor layer, in the nanowire, or at their interface^186,199,200^. Materials science efforts nearly perfected the superconductor–nanowire interface, leading to hard induced superconducting gaps, i.e., without any in-gap conductance, measured in both InAs and InSb nanowires^187,192−194,199,201^ (Figure 6f,g). On average, the induced superconducting gap of Al in InAs and InSb ranges between 0.19 and 0.24 meV^186,187,199,201^, comparable to the bulk Al superconducting gap ≈0.2 meV. It has been reported that the Al superconducting gap increases as a function of decreasing Al film thickness.^202^ Generally, larger superconducting gaps with a higher critical magnetic field and critical temperature are beneficial since they do not limit the energy parameter space to access topological superconductivity.

As pointed out by recent proposals, the nearly identical values of induced and parent superconducting gaps insinuate that material science efforts were perhaps excessively successful in obtaining clean, superconductor–nanowire interfaces that result in an overly strong superconducting-nanowire coupling^205−207^. This overly strong coupling overwhelms the intrinsic properties of the nanowire, yielding an induced superconducting gap nearly identical to that of the superconductor. These results bring into question whether the properties of the nanowire are at all relevant and, in turn, whether a topological phase transition can be achieved in the presence of this strong coupling. These proposals recommend adding a thin insulating layer between the superconductor and the nanowire to reduce the coupling strength. To this date, transport experiments aimed at measuring the induced gaps in the presence of an insulating interface layer in superconductor-insulating layer-nanowire hybrids have not been explored. Despite recent materials science demonstrating a tunable, epitaxial CdTe layer (as the thin insulating layer, i.e., tunnel barrier) between InSb nanowires and largely epitaxial Al,^208^ tunability of the coupling has not been measured and its success is yet to be determined.

Addressing the Majorana conductance peak height is rather complicated, especially since a lot of other phenomena yield (quantized) zero bias conductance peaks resembling the Majorana signature, such as the presence of quantum dots, nonhomogeneous pairing potentials, the Kondo effect, or Andreev bound states^209−212^. Andreev bound states develop through Andreev reflection, a process that occurs when an electron with an energy less than the superconducting gap enters the superconductor and is reflected as a hole, such that a Cooper pair is formed and a net charge of two electrons is transferred to the superconductor.^213^ The pinning of these Andreev bound states close to zero energy makes it experimentally challenging to distinguish them from topological Majorana zero-energy peaks^213,214^. After all, the multitude of phenomena that manifest zero bias peaks cast doubt on whether zero bias peaks measured thus far are actually related to Majoranas and call for novel device fabrication methods, materials science breakthroughs, and measurement techniques to conclusively dismiss non-Majorana peaks.

Besides the common N-NW-S spectroscopy technique to detect Majorana zero bias peaks, a less direct approach relies on Coulomb blockade spectroscopy on a so-called Majorana island geometry.^215^ A proximity-induced superconducting segment (island) is separated from metal electrodes at both ends of the nanowire via tunnel barriers (Figure 6h). Tunneling onto the island depends on the charging energy compared to the size of the induced superconducting gap. In particular, if the charging energy of the island is smaller than the induced superconducting gap then a superconducting ground state on the island is preferred, leading to a Coulomb blockade with a gate-voltage period of 2*e*, since there are no states available for a single electron to tunnel onto the island (Figure 6i). In the presence of Majoranas at both ends of the superconducting island, Coulomb blockade becomes 1*e* periodic, as single electrons can be teleported coherently through the Majorana states^215,216^. While 2*e*-1*e* transitions have been measured in InAs and InSb nanowire hybrids using various superconductors^193,194,215,217,218^, the Coulomb blockade spectroscopy method similarly struggles with unambiguously excluding non-Majorana states, such as Andreev bound states, that can give rise to 2*e*-1*e* oscillations.

While the search for Majoranas in nanowires faces a meandering road ahead, research in hybrid nanowire devices has opened up avenues for investigating novel phenomena and devices including Josephson junctions^187,196,219−222^ and Cooper pair splitters^223−225^.

## 3 Group IV Nanowires

Group IV semiconductors such as silicon (Si), germanium (Ge), and their alloys have been dominating semiconductor devices for more than half a century, rendering “Si” almost synonymous to the semiconductor-industry, a prominent example of which is Silicon Valley, known for its specialization in silicon transistors and integrated circuits. Because Si is and has been in every integrated circuit since the 1960s, there is extensive knowledge about it, ranging from growth, impurity doping, surface passivation through to contacting, thereby facilitating rapid progress in device development and fabrication. Among the main drivers for Si technology are the special features of this material, such as its abundance, low cost, and most importantly, its exceptionally high-quality native oxide.^226^ Silicon dioxide (SiO_2_) is chemically stable and provides an excellent insulator with superior dielectric, electrical, and mechanical properties.^227^ In particular, it can be grown on Si with an abrupt interface as a conformal layer, free of holes down to a few monolayers with very few electrically active defects^227,228^.

These unique properties of Si, most notably its oxide, resulted in Si dominating the semiconductor industry with no other semiconductor in contention. Nevertheless, Si has a relatively low carrier mobility. A common approach to enhance the carrier mobility, of both electrons and holes, is by strain engineering.^229^ Strain is often induced by lattice-mismatched film growth and can be engineered to reduce the symmetry of the silicon crystal, giving rise to band splitting and band warping, thus yielding a lower effective carrier mass and in turn a higher carrier mobility.^229^ Unlike Si, the carrier mobility in Ge is higher. The effective mass of holes in Ge is especially low resulting in a high mobility comparable to or even higher than in III–V semiconductors. In fact, Ge has the highest hole mobility of any semiconductor at room temperature.^226^ State-of-the-art two-dimensional hole gases in strained Ge/Si heterostructures show mobilities up to 1.5 × 10^6^ cm^2^/(V s) at low temperatures.^230^

In nanowire form, the synthesis of Si nanowires is as old as the 1960s.^62^ Similar to the bulk form, techniques to improve and tune the nanowire properties are adopted, including growth of various heterostructure configurations (axial and radial), strain, and doping. These techniques have rendered Si nanowires compelling for a range of applications from photovoltaic applications^231,232^, batteries,^233^ biosensors,^234^ through to field-effect transistors^235−238^. Since this review focuses on nanowire applications in the context of quantum information processing, the interested reader is referred to excellent reviews on the growth, properties, and applications of Si and Si_1–*x*_Ge_*x*_ nanowires^239−241^.

Si_1–*x*_Ge_*x*_ nanowires are particularly interesting for a number of quantum devices, most notably spin-based devices. The superior spin properties of Si_1–*x*_Ge_*x*_ stems from the absence of nuclear spin in the most abundant even-number isotopes of Si and Ge, in contrast to III–V materials. As a consequence, the carrier spin lifetime (coherence time) can be much longer^242−244^ compared to III–V materials where surrounding nuclear spins interact with the electron spin, leading to decoherence. Moreover, spin–orbit coupling can be significantly increased by lattice strain, using a core–shell nanowire configuration.^245^ The combination of long coherence time and strong spin–orbit coupling makes Ge–Si core–shell nanowires an extremely interesting system for spin qubits.^226^ Ge–Si core–shell nanowires have also been proposed for the detection and manipulation of Majorana zero modes or parafermions^246,247^ because of the predicted high spin–orbit energy in Ge–Si core–shell wires.

The core–shell configuration has several advantages. The shell can passivate the nanowire surface, and thus reduce surface scattering^248,249^. In particular, since surface scattering is known to be a dominant limiting factor for carrier mobility in nanowires. In addition to passivation, the core–shell geometry also enables remote doping, such that dopants can be incorporated in the shell and free carriers are donated to the transport channel in the core, yielding an increase in mobility.^238^ Another feature of the core–shell geometry is that strain can be introduced by growing a shell that is lattice mismatched with the core. The Ge–Si core–shell configuration is commonly used since Ge and Si have a 4.2% lattice mismatch, and this can be used to tune the strain level^250−253^. The small nanowire dimensions and shape allow for inducing high strain levels in both core and shell that are tunable by both the core diameter and the shell thickness. The advantages of the Ge–Si core–shell system have led to its widespread use in quantum transport studies.

Studies on Si and Ge heterostructures show that the conduction and valence band offsets between Si and Ge depend strongly on strain. The band offsets have been calculated for different Si_1–*x*_Ge_*x*_*x* compositions and for different heterostructure configurations^254−257^. For all compositions, the valence band edge is higher in the Ge-rich layer with a large offset in the range of 0.1–0.6 eV, providing a large confinement potential for holes. By contrast, the conduction band offset is very sensitive to the composition and strain. In particular, for all compositions the band offsets are very small (±20 meV) and can switch sign depending on the strain level. Therefore, in a Ge–Si core–shell nanowire, the holes are strongly confined in the Ge core, rendering Ge–Si core–shell nanowires particularly suitable to explore hole physics. The p-orbital symmetry of holes gives rise to a total angular momentum of *J* = 3/2, which results in an unusually large Rashba type spin–orbit interaction that can be tuned by electric fields and lattice strain.^245^ In addition, the p-type symmetry of holes suppresses hyperfine interactions, leading to long spin-hole lifetimes.^226^ Increasing the strain leads to an increase of the sub-band level splitting and thus increases the energy scales. Spin–orbit energies are in the ≥1 meV range, which is much higher compared to other semiconductors^245,258^. Furthermore, the strong spin–orbit interaction can induce a helical ground state in the presence of a magnetic field, in which holes with opposite spin move in opposite directions. Similar to InAs and InSb nanowires, when coupled to an s-wave superconductor Majorana zero modes are expected to emerge at both ends of the Ge–Si core–shell nanowire.^246^

### 3.1 Synthesis of Group IV Nanowires

The use of group IV nanowires in various devices requires a high crystalline quality (with minimal crystal defects). Furthermore, the ability to grow them thin is crucial since most transport studies rely on the one-dimensional behavior of the nanowire, and since the effective masses are relatively high, small diameters are required. Lastly, it is important to control their impurity doping. There are two reported methods to fabricate group IV nanowires: top-down by using lithography and etching^259−261^ and bottom-up, most commonly using the VLS method. In the top-down approach, controlled oxidation and oxide removal is used to further reduce the nanowire diameter^262,263^. This technique has resulted in nanowires with sub 10 nm diameter nanowires. As for the crystal structure and doping density, they are determined by the substrate from which the wires are carved. Similar approaches are being used in the semiconductor industry to realize gate-all-around architectures.^264^

Besides the prevailing VLS technique, a unique bottom-up approach enabled the epitaxial growth of in-plane Ge nanowires, referred to as “Ge Hut wires”.^265^ The hut wires are fabricated by epitaxially depositing a Ge wetting layer on a Si(100) substrate.^265^ After thermal annealing, the metastable wetting layer evolves into Ge wires with uniform lateral dimensions due to the lattice strain. These resulting self-assembled wires are roughly 100–1000 nm long and about 2 nm high, exhibiting a triangular cross-sectional shape with a base of about 15 nm. These nanowire are composed of Ge_1–*x*_Si_*x*_ with a Ge concentration ≥65% as a result of intermixing during the annealing process. After formation of the Ge lines, they can be capped with a silicon layer at relatively low temperature, resulting in fully strained Ge. One advantage of this approach is that the wires are in-plane, i.e., parallel to the substrate, thus facilitating device fabrication as compared to out-of-plane nanowires. Moreover, this approach dispenses with the catalyst, thus circumventing the incorporation of undesired impurities in the nanowire. This approach, however, has little to no control on the position and the density of these Ge nanowires.

The majority of group IV nanowires used for quantum transport measurements have been grown using the widespread VLS technique. The main challenge for group IV nanowires as well as their core–shell equivalent is the incorporation of catalyst atoms in Si^266,267^. Generally, the growth of Si and Ge nanowires relies on a gold catalyst particle. In part, because gold has a simple phase diagram with these elements with a low eutectic point facilitating growth at relatively low temperatures.^268^ Nevertheless, a problem associated with gold as a catalyst particle is the high solubility and diffusion rate of gold in Si.^267^ It has been shown that gold diffuses throughout the whole nanowire during growth and gold atoms are trapped in the nanowire.^266^ Critically, gold atoms act as trap states,^269^ and they induce side wall roughening, possibly deteriorating the electronic properties.^270^ A way to avoid gold incorporation on the nanowire surface or in the shell in a core–shell configuration is by using a diffusion-blocking layer top segment^270,271^.

In the core–shell configuration, lattice strain between the Ge core and the Si shell may lead to the formation of misfit dislocations. This type of defect is undesirable as it can scatter charge carriers. Defect-free core–shell nanowires can be achieved by reducing the lattice mismatch between core and shell by growing Si_1–*x*_Ge_*x*_ shells on Ge cores,^252^ alternatively by growing Si shells on Si_1–*x*_Ge_*x*_ cores.^272^ While strain-induced misfit dislocations can be reduced by using amorphous shells to passivate the nanowire surface,^237^ for some applications it is beneficial to conserve strain, such as spin-based devices, where compressive strain has been predicted to dramatically increase the spin–orbit energy in Ge cores^245,258^.

As mentioned in the section on III–V nanowire growth, SAE (or SAG) would enable making more complex nanowire device designs and increasing scalability. Note that compared to InAs and InSb, much smaller dimensions are required for Ge/Si due to the higher effective mass of the carriers in Si and Ge. Although selective-area growth has been used for the growth of large-areas of Si and Ge, it is unexplored for the growth of Ge/Si core/shell nanowire structures. In a first report on the SAG of Ge nanowires on Si(100),^273^ the Si surface was conditioned by As to create an oxide-free surface. The resulting wires have a 80 nm diameter, and the remaining challenge is to realize smaller dimensions to avoid inelastic strain relaxation by the creation of crystal defects.

### 3.2 Transport in Group IV Nanowires

For electronic transport studies it is essential that the metallic leads contacting the nanowire are Ohmic contacts, which remain highly transparent down to low temperatures. Ohmic contacts have been fabricated with titanium/gold contacts for Si^237^ and with palladium, titanium/palladium, or nickel contacts for Ge wires^236,238,274,275^. Essentially, most metals on Ge show Fermi-level pinning near the valence band,^276^ simplifying the fabrication of Ohmic contacts and dispensing with the high-temperatures annealing steps required for local doping. More advanced contact schemes include the in-diffusion of the contact metal into the nanowire channel forming silicides or germanides. Silicidation is a thermally activated process used in conventional transistors to make low-resistive contacts. In general, M/A phases (with M = Ni, Pt, Co, Al, etc. and A= Si, Ge) can be formed, as demonstrated for Ni/Si^277−280^, Ni/Ge,^281^ and Al/Ge^282^ contacts.

Basic electronic properties of nanowires can be determined using field-effect-transistor devices, as pointed out in section 2.2.1. Hole mobilities in the range of 100–600 cm^2^/(V s) have been measured in unpassivated Ge nanowires at room temperature^236,283^. Higher mobilities were obtained in modulation doped Ge–Si_0.45_Ge_0.55_ core–shell nanowires with peak mobilities reaching 700–1800 cm^2^/(V s) at 77 K.^238^ While the measured hole mobilities remain slightly lower in Ge–Si core–shell nanowires compared to pure bulk Ge (1900 cm^2^/(V s) at room temperature), these mobility values are extremely high considering the high carrier concentration of 10^19^ cm^–3^.^271^ Mobility in Ge–Si core–shell nanowires was shown to also depend on the nanowire orientation. In particular, [110]-oriented wires exhibit substantially higher hole mobility, compared to common [111]-oriented wires, with values reaching 4200 cm^2^/(V s) at 4 K.^271^

**Figure 7:**
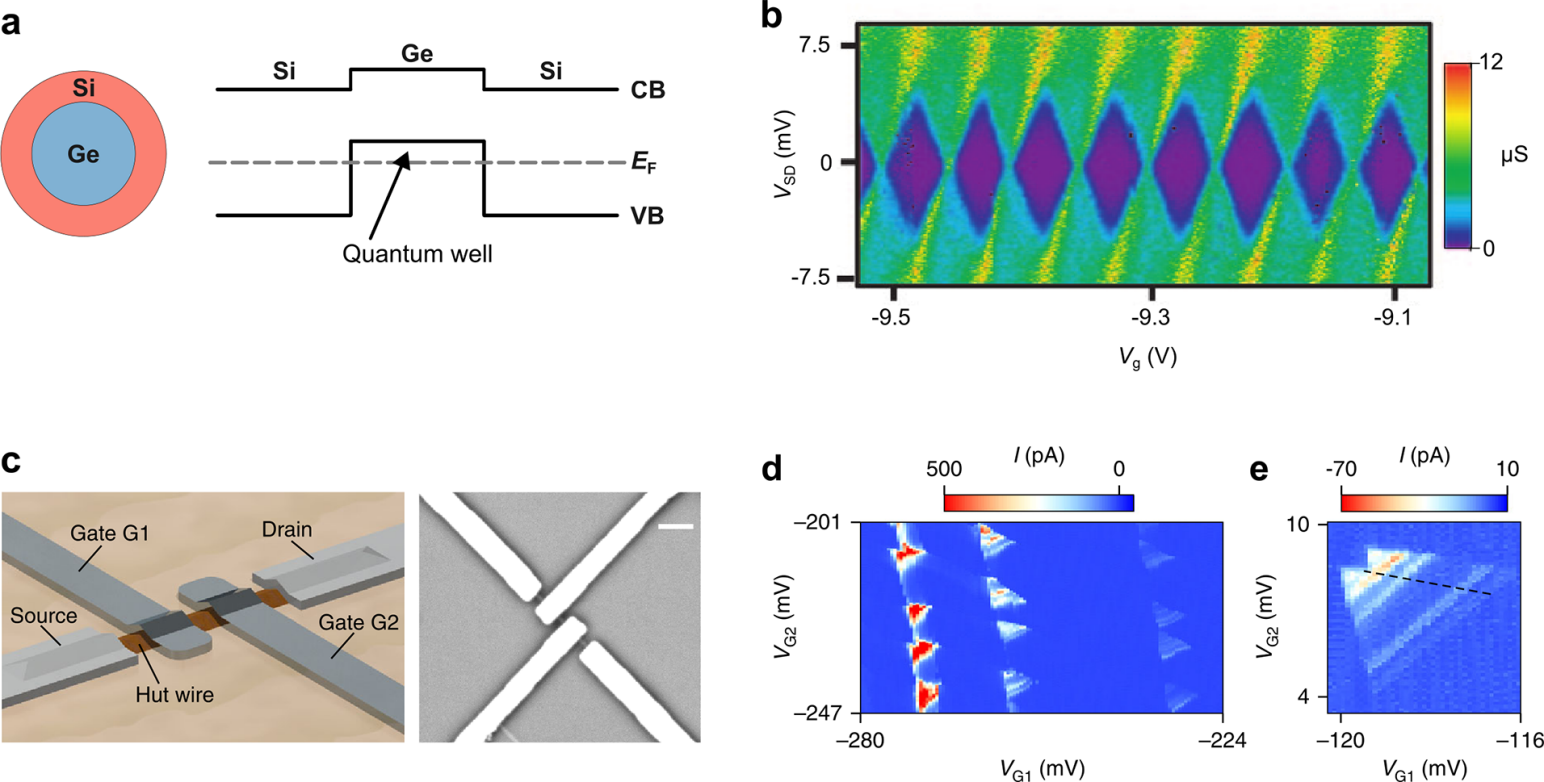
Ge–Si devices. (a) Schematic representation of the cross-section of a Ge–Si core–shell nanowire along with the band diagram for this heterostructure. The Fermi level, *E*_F_, lies inside the Si band gap and below the Ge valence band edge.^284^ (b) Differential conductance *G* = d*I*/d*V*_SD_ vs *V*_SD_ and *V*_g_ for a 10 nm core diameter Ge–Si nanowire device (*T* = 1.5 K, *V*_SD_ = 0.5 mV, and *L* = 112 nm) exhibits well-defined Coulomb diamonds in line with transport through a single-electron transistor. The Ge–Si device is prepared with Si tunnel barriers through which electrons have to tunnel.^284^ (c) A schematic (left) and a SEM image (right) of a Ge hutwire double quantum dot (DQD) device contacted with source and drain electrodes and covered by two top gates (G1, G2). Scale bar is 200 nm.^285^ (d) Stability diagram of a DQD showing characteristic bias triangles at *V*_SD_ = 2 mV.^285^ (e) Zoom-in on a pair of bias triangles at *V*_SD_ = −2 mV of two bias triangles from a second device. Due to the fairly low mutual capacitance, the triangles are merged already at relatively low bias voltages. The base of the triangle marks current flowing through the ground states. The parallel lines within the triangles denote transport through excited states. Energy level separations of up to ≈1 meV are measured.^285^

Quantized conductance has been measured in ultrathin (15–5 nm) Ge–Si core–shell nanowires in channel lengths up to 500 nm, indicating ballistic hole transport^284,286,287^, with mean free paths in the order of 170–540 nm at low temperatures and exceeding 170 nm at room temperature.^288^ Moreover, a sub-band spacing of 20–25 meV between the first and the second sub-band is extracted.^284^ These experiments demonstrate that a one-dimensional hole gas can be achieved in high-quality bottom-up grown nanowires. One-dimensional hole gas systems can be engineered in Ge–Si core–shell nanowires, since there is a rougly 500 meV valence band offset between the Ge core and Si shell in this heterostructure, leading to the accumulation of free holes in the Ge channel when the Fermi level is below the valence band edge of the Ge core, as shown in Figure 7a.^284^ Using the same Ge–Si core–shell heterostructure, a single electron transistor has been fabricated. In particular, by fabricating contacts without contacting the Ge channel, carriers need to tunnel through the nonconductive Si shell, resulting in a barrier in transport measurements at low temperatures.^284^ This 112 nm-long Ge–Si device exhibits periodic Coulomb blockade diamonds in line with single-electron transport (Figure 7).

Furthermore, quantum dots as small as 25–30 nm have been realized in a 3–8 nm diameter Si nanowire by in-diffusion of Ni from the contacts.^289^ In this study, the quantum dot could be depleted to the very last hole. Similarly, depletion to the very last hole has been achieved in Ge–Si core–shell nanowires^290,291^. Crucially, the experimental realization of gate-tunable quantum dots, where the charging energy can be precisely modulated, enables the probing of *g*-factors, spin states, Pauli spin blockade, and charge sensing^274,275^.

As mentioned in Section 2.2.2, the *g*-factor determination requires a magnetic field (*B*-field) to lift the spin degeneracy. A *g*-factor in the range of 1.0–2.2 is obtained for quantum dots in Ge–Si core–shell nanowires, a range which is lower than that of unperturbed holes in Ge, and close to a free spin-1/2 particle^243,292^. These values are likely attributed to strong confinement in the quantum dots as well as mixing between heavy- and light-holes. A *g*-factor up to 8 has been extracted from spin-blockade measurements.^293^ This high *g*-factor could be due to a large diameter nanowire (20–30 nm Ge core and 2 nm Si shell). Rotating the magnetic field exhibits a *g*-factor anisotropy, where the highest values *g* = 2.1–2.7 are obtained for a *B*-field perpendicular to the nanowire axis and the lowest *g* = 0.2 for a parallel field.^274^ This anisotropy depends on the nanowire dimensions (confinement potential) and can be tuned by electric fields^294,295^. The electric-field tunability is caused by an enhanced Rashba-type spin–orbit interaction because of the mixing of heavy and light hole states. This enhanced spin–orbit interaction (SOI) is referred to as direct Rashba spin–orbit interaction (DRSOI) and is predicted to be 10–100 times stronger compared to the standard Rashba SOI^245,258,274^, allowing fast coherent spin manipulation.

Single and double quantum dots have also been obtained in Ge hut-wires (Figure 7c–e), where the Pauli spin blockade has been demonstrated in double quantum dots^285,296^. By using electric-dipole spin resonance, a single hole spin could be addressed. Rabi oscillations with frequencies approaching 140 MHz were shown, and a lower bound for the dephasing time, *T*_2_, of 33 ns was estimated.^285^ Coherent control over the hole spin state was shown using periodic square pulses with average dephasing times exceeding 130 ns.^285^ Large anisotropies in *g*-factors also manifest in Ge hut wires, with *g*-factors as high as 4.4 extracted.^297^ These experiments pave the way toward scalable spin qubits.

#### 3.2.1 Group IV Nanowire Hybrids

While most experimental research is focused on nanowire hybrids involving III–V semiconductors, most notably, InAs and InSb, group IV nanowires offer an appealing alternative because of their unique properties, such as their strong SOI and tunable *g*-factors.

A Josephson junction based on a Ge–Si core–shell nanowire and aluminum leads with a source drain spacing of 100 nm showed critical currents greater than 100 nA.^298^ Moreover, signatures of higher order Andreev reflections have been obtained in addition to nearly ideal *I*_c_*R*_N_ product values (*I*_c_ the critical current, *R*_N_ the normal state resistance), implying that the contacts are highly transparent (80% transmission probability), and the channel allows for coherent transport. The demonstration of high-quality proximitized Ge–Si core–shell nanowires along with a DRSOI render one-dimensional hole gases in Ge–Si nanowires a potential candidate for the realization of topological superconductivity^246,290,299^.

Ge–Si core–shell nanowires constitute a very promising platform for the fabrication of spin qubits and for realizing topological quantum circuits.^226^ The growth of selective area Ge nanowires on Si substrates would enable better control of the position and the dimensions of the wires. Position control is not only important for the realization of a scalable technology but also for creating novel devices. In particular, two wires connected by a superconductor at a distance shorter than the superconducting coherence length are predicted to host crossed Andreev reflection processes, effectively splitting a Cooper pair and potentially attaining parafermions.^247^ Similar to Majorans, parafermions can be braided, yielding protected gate operations.

To further increase spin–orbit interaction in Ge nanowires is to alloy them with tin^300,301^, since tin is a heavier element than Ge and will thus naturally increase spin–orbit interaction. Another important research direction would be to integrate superconductors epitaxially on Ge–Si nanowires as has been shown for III–V nanowires. For Ge–Si nanowires, group IV superconductors Sn and Pb represent potential candidates, since they would not introduce impurity dopants to the Ge–Si materials system in addition to their superior superconducting properties, including large critical temperatures and fields. Combining these properties with the predominant hole, quasiparticles in Ge–Si nanowires could pave the way to novel Majorana devices.

## 4 Summary and Outlook

III–V nanowires, most notably InAs and InSb, have played a prominent role in the ongoing search for topological states in superconductor-semiconductor hybrids. Their unique properties should offer the necessary requirements to host topological phases, but these have not been unambiguously demonstrated. Recently, indications for the presence of a small topological gap in InAs has been reported.^307^ Moreover, taking advantage of the nanowire properties instigated the branching of various research directions, from single-electron spin physics to ballistic transport through to Kitaev chains. Comparably, Si–Ge nanowires have granted access to (single) hole physics and compromise a viable route for spin qubits, not only because of their long-lived spin coherence but also because of their relatively straightforward integration on the existing Si platform.

These advances and access to new insights call for studying alternative materials with better properties: stronger spin–orbit coupling, larger *g*-factors, higher mobility, and possibly less disorder. The heavy-element lead telluride (PbTe) emerges as a suitable semiconductor due to its strong spin–orbit coupling and large anisotropic Landé *g*-factor^308^ (see Table 1). Importantly, PbTe possesses an extremely large static dielectric constant, which shields it from fluctuations caused by charge impurities and crystal defects, effectively reducing disorder^309,310^. While somewhat a nascent material in the nanowire community, PbTe nanowires have appeared in a few recent publications encompassing VLS out-of-plane PbTe nanowires^311,312^ and in-plane PbTe nanowire networks^313−315^ with a low-temperature phase coherence length exceeding 21 μm.^314^

**Table 1:** Material Properties Relevant for Transport Experiments^a^

	μ (cm^2^/(V s))	*g*-factor	*E*_SO_ (meV)	ε_r_
InAs	1.0 × 10^4^^85,98,113,114^	2–18^47,141,148,149^	0.2–1^167^	15.2*^302^
InSb	3.0 × 10^4^^58,119−121^,	38–55^49,120,137^	0.02–0.14^167^	16.8*^302^
Ge/Si	0.7–4.2 × 10^3^^238,271^	1–8^292−297^	≈1^★^^245^	16.2*^302^
PbTe	1.0 × 10^6^*^303^	66*^304^	0.17–0.6^305^	400–900*^306^

a Relevant parameters include carrier mobility μ, *g*-factor, spin–orbit energy *E*_SO_, and dielectric constant ε_r_. Given values are experimentally obtained from nanowire devices, whereas values denoted by * are bulk values and those denoted by ★ are theoretically predicted. Quoted mobility values are for electron mobility, except for Ge/Si, where it is hole mobility.

Another evolving research field encompasses nanowires made of materials categorized as topological insulators (TIs) and topological crystalline insulators (TCIs). Topological insulators are a new class of materials which have a finite, inverted band gap in their bulk, and a gapless state on the surface with linear energy dispersion^8,316,317^. The existence of these surface states is not due to a “local” distortion of the system (e.g., Fermi-level pinning on dangling bonds), but due to a global, nontrivial topology of the band dispersion, which can be described by a topological invariant (often referred to as “*Z*_2_ invariant”). Since these surface states are a global material property, they are exceptionally robust against local perturbations and, therefore, an interesting system to study quantum effects. From the large number of topological materials^318−320^, the most common ones are found in the V–VI family of crystals, such as Bi_2_Se_3_, bismuth telluride (Bi_2_Te_3_), and antimony telluride (Sb_2_Te_3_)^321−324^. Moreover, the recently discovered topological crystalline insulators share many similarities with conventional TIs, in particular the presence of surface states. The main difference between TIs and TCIs lies in the surface-state protection. Whereas time-reversal symmetry protects TI-surface states, their TCI counterparts are protected by the mirror symmetry of the rock-salt crystal, and thus the surface states only exist on the high-symmetry facets {001}, {110}, and {111} of the crystal. The different protection mechanisms imply that the surface states of TIs (time reversal symmetry) are not robust against a magnetic field, in contrast to TCI surface states (crystal symmetry). Specific compositions of rock-salt lead tin selenide telluride (Pb_1–*x*_Sn_*x*_Se_1–*y*_Te_*y*_) are a TCI^325−327^. The main interest in the nanowire geometry is driven by the enhanced surface-to-volume ratio compared to bulk crystals, which should help suppress the parasitic bulk background conductance present in this material class. One of the challenges with TI/TCI nanowires resides in the difficulty to prove the TI/TCI origin of the surface conduction, despite the existence of rather straightforward experiments to probe the surface character of conductance in a nanowire. The field of TI/TCI nanowires is progressing and still requires the development of advanced devices to address open challenges to grant entry to novel quantum phenomena.
